# The giant butterfly-moth *Paysandisia archon* has spectrally rich apposition eyes with unique light-dependent photoreceptor dynamics

**DOI:** 10.1007/s00359-018-1267-z

**Published:** 2018-06-04

**Authors:** Primož Pirih, Marko Ilić, Jerneja Rudolf, Kentaro Arikawa, Doekele G. Stavenga, Gregor Belušič

**Affiliations:** 1 Department of Evolutionary Studies of Biosystems, SOKENDAI The Graduate University for Advanced Studies, Shonan International Village, Hayama, 240-0115 Kanagawa Japan; 2 Department of Artificial Intelligence, University of Groningen, Nijenborgh 9, 9747 AG Groningen, The Netherlands; 3 Department of Biology, Biotechnical faculty, University of Ljubljana, Večna pot 111, 1000 Ljubljana, Slovenia; 4 Sars International Centre for Marine Molecular Biology, University of Bergen, Thormøhlensgt. 55, 5006 Bergen, Norway; 5 Department of Computational Physics, Zernike Institute for Advanced Materials, University of Groningen, Nijenborgh 4, NL, 9747AG Groningen, The Netherlands

**Keywords:** Palm borer moth, Compound eye, Spectral sensitivity, Lepidoptera, Phylogeny

## Abstract

The palm borer moth *Paysandisia archon* (Burmeister, 1880) (fam. Castniidae) is a large, diurnally active palm pest. Its compound eyes consist of ~ 20,000 ommatidia and have apposition optics with interommatidial angles below 1°. The ommatidia contain nine photoreceptor cells and appear structurally similar to those in nymphalid butterflies. Two morphological ommatidial types were identified. Using the butterfly numbering scheme, in type I ommatidia, the distal rhabdom consists exclusively of the rhabdomeres of photoreceptors R1–2; the medial rhabdom has contributions from R1–8. The rhabdom in type II ommatidia is distally split into two sub-rhabdoms, with contributions from photoreceptors R2, R3, R5, R6 and R1, R4, R7, R8, respectively; medially, only R3–8 and not R1–2 contribute to the fused rhabdom. In both types, the pigmented bilobed photoreceptors R9 contribute to the rhabdom basally. Their nuclei reside in one of the lobes. Upon light adaptation, in both ommatidial types, the rhabdoms secede from the crystalline cones and pigment granules invade the gap. Intracellular recordings identified four photoreceptor classes with peak sensitivities in the ultraviolet, blue, green and orange wavelength regions (at 360, 465, 550, 580 nm, respectively). We discuss the eye morphology and optics, the photoreceptor spectral sensitivities, and the adaptation to daytime activity from a phylogenetic perspective.

**Electronic supplementary material:**

The online version of this article (10.1007/s00359-018-1267-z) contains supplementary material, which is available to authorized users.

## Introduction

The palm borer moth *Paysandisia archon* (Burmeister, 1880) (Lepidoptera: Castniidae) is a large moth native to Uruguay and Argentina. Its caterpillars live in galleries in palm tree trunks. The recent introduction to Europe has had a devastating effect on the decorative palm trees around the Mediterranean Sea and may potentially cause severe problems to the date production in North Africa and the Levant. The adult male and female *Paysandisia* are diurnally active (Sarto i Monteys et al. 2016; Frérot et al. 2017). The male emits short-range pheromones and is territorial. As the female probably does not produce long-range pheromones, to initiate mating, a male must first see a female before it starts displaying its colours at close range, suggesting that the large compound eyes play an important role in intraspecific recognition (Sarto i Monteys et al. 2012; Frérot et al. 2013; Quero et al. 2017; review: Sarto i Monteys et al. 2016).

The compound eyes of *Paysandisia* have a conspicuous pattern of pseudopupils, resembling the pseudopupil pattern of the apposition eyes of pierid and nymphalid butterflies (Stavenga 1979), thus indicating that also *Paysandisia* has apposition eyes. *Paysandisia* belongs to the family Castniidae, part of the superfamily Cossioidea, which groups together with Zygaenidae (burnets). The two superfamilies are placed in the clade Apodytrisia, either just outside or in the beginning of the subclade Obtectomera (Mutanen et al. 2010; Heikkilä et al. 2015; Mitter et al. 2017). The day-flying apodytrisian moths have apposition eyes, e.g. members of the families Sesiidae (Eby et al. 2013), Epicopeiidae and Zygaenidae (Yagi and Koyama 1963). In the order of Lepidoptera, however, the majority of species is nocturnally active. Their superposition eyes provide a higher sensitivity in low-light conditions (Land and Nilsson 2012). A recent comparative morphological study of the microlepidopteran families basal to Apodytrisia has postulated an intermediate eye morphology as an adaptation of minute-sized eyes to nocturnal activity (Fischer et al. 2012, 2014). A similar morphology where the proximal rhabdom is thick, while the thin distal rhabdoms reach the dioptric apparatus, has also been confirmed with ultrastructure in comparatively larger eyes of some moth families, e.g. in Tortricidae (Satoh et al. 2017), Pyralidae (Horridge and Giddings 1971) and Crambidae (Belušič et al. 2017) and may be present in some other groups (Yagi and Koyama 1963). Most “true butterflies”, the Papilionoidea, have apposition eyes with thin rhabdoms, with the exception of the diurnal Hesperiidae (Horridge et al. 1972; Shimohigashi and Tominaga 1986) and nocturnal Hedylidae (Yack et al. 2007), which both have superposition eyes.

A requirement of the superposition eye design is that the dioptric apparatus has afocal (telescopic) optics (Nilsson 1989). The afocal optics has been shown in the apposition eyes of some butterfly species (Nilsson et al. 1988). Detailed functional studies on the visual system of moths have mostly been limited to a few species with superposition eyes from a limited number of families. Spectrophotometry on isolated retinae demonstrated the presence of three classes of photoreceptors, with visual pigments absorbing maximally in the ultraviolet (UV), blue (B), and green (G) wavelength range, in agreement with spectral sensitivities measured by electrophysiological recordings (e.g. Hamdorf et al. 1973; White et al. 1983; reviewed in Briscoe and Chittka 2001). For the tobacco hawk moth *Manduca* (Sphingidae), optical and electrophysiological results were corroborated with the expression pattern of three opsins (White et al. 2003). Using microspectrophotometry and light-induced ultrastructural changes, a tiered rhabdom with nine retinula cells was shown in the saturniid moth *Antheraea*, where the two distal cells are blue or UV sensitive, the medial cells are green sensitive and the basal cell contains a blue rhodopsin (Langer et al. 1986). Evidence for four photoreceptor classes in a superposition eye was obtained in the African armyworm *Spodoptera* (Noctuidae) using spectrometry (Langer et al. 1979; Meinecke and Langer 1984) and in the corn borer moth *Ostrinia* (Crambidae) by intracellular recordings (Belušič et al. 2017). To our knowledge, there are no functional studies of the visual system in apodytrisian moths, so one has to rely on the interpretations of earlier collections of histological data (e.g. Ehnbom 1948; Tuurala 1954; Yagi and Koyama 1963).

The structural and functional aspects of butterfly (papilionoid) eyes have been studied extensively. The emerging comparative picture is that butterfly compound eyes have three ommatidial types, with each ommatidium containing nine photoreceptor (retinula) cells (Arikawa and Stavenga 1997; Qiu et al. 2002; Ogawa et al. 2013; Chen et al. 2016; reviewed by: Stavenga and Arikawa 2006; Arikawa 2017). The butterfly (B) numbering scheme maps to the dipteran (D) numbering scheme in the following manner: B1–2 → D7, B3–8 → D1–6, B9 → D8 (Friedrich et al. 2011; Arikawa 2017). The number of spectral receptor classes varies among different butterfly species. Intracellular recordings in some nymphalids identified a basic set of spectral receptors with three visual pigments (Kinoshita et al. 1997), which appears to be based on three opsins (Briscoe et al. 2003; Briscoe and Bernard 2005). The photoreceptor spectral sensitivities can be modified by multiple opsin expression in one cell (Kitamoto et al. 1998; Ogawa et al. 2012) and by screening pigments acting as spectral filters (Arikawa et al. 1999; Stavenga et al. 2001; Stavenga 2002; Qiu et al. 2002; Zaccardi et al. 2006; Stavenga and Arikawa 2011). The papilionoid *Papilio xuthus* employs both mechanisms and appears to have five opsins that serve as the basis for six spectral receptor classes (Arikawa 2017). The modification of spectral sensitivity by screening pigments has been recently reported also for a tortrix moth with an intermediate eye type (Satoh et al. 2017).

Here we report our investigations on the compound eyes of *Paysandisia*. Intracellular electrophysiological recordings of the spectral sensitivities of the photoreceptors revealed the presence of four photoreceptors classes, with maximal sensitivities in the UV, blue, green and orange wavelength range, respectively. Anatomy, using light and electron microscopy, identified ommatidia with nine photoreceptor (retinula) cells. Based on rhabdom structure, the ommatidia were classified into two distinct types. *Paysandisia* shows active light adaptation mechanisms, in which the rhabdom secedes from the crystalline cone together with extensive pigment migrations. We discuss the possible spectral discrimination ability for visual communication and the apposition eye design of *Paysandisia* in relation to the phylogeny of higher Lepidoptera.

## Materials and methods

### Animals

Pupae and living adults were obtained from the Palm Protect consortium partner CIRAD/CSIRO in Montpellier, France. The animals were transferred to Slovenia under permit no. 3430 119/2012/2 issued by the Phytosanitary administration of the Slovenian Ministry of Agriculture and Environment. The anatomical studies were done on five animals, extracellular recordings on three animals and intracellular recordings on six animals. There were no apparent differences in the results between males and females.

### Anatomy

Isolated retinae were treated with standard procedures (pre-fixed for 3 h in 4% paraformaldehyde and 3.5% glutaraldehyde, post-fixed for 90 min in 0.1 M OsO_4_ in 0.1 M Na cacodylate, pH 7.4, dehydrated in acetone series, and then embedded in EPON resin). Dissections were performed around 9 a.m. Due to the large size of the eye, delayed penetration of the fixative caused swelling and mechanical stress, resulting in tissue tearing in the distal eye parts in some preparations. Semi-thin sections (1–2 µm) for light microscopy were cut on an Ultracut microtome (Leica, Mannheim, Germany) with a glass knife. Ultrathin sections were made on the same microtome with a diamond knife and observed with an H-7650 transmission electron microscope (Hitachi, Tokyo, Japan). To study the effects of light adaptation, moths were either exposed to indoor fluorescent lighting, put for 1 hour into complete darkness, or kept in sunlit cages prior to fixation. Thus, the retinae were either dark adapted, intermediately adapted (adapted to intermediate, indoor light), or light adapted (adapted to bright light). Dissection of the eyes of dark-adapted animals was performed under NIR light (850 nm), using a microscope with a NIR-sensitive USB camera (Dino Lite AM4115-FIT, AnMo Electronics, New Taipei City, Taiwan).

The presence/absence of the tapetal reflection (eyeshine) was inspected with an epi-illumination telemicroscope (Stavenga 2002). The curvature of the compound eye was measured in a living, immobilized specimen, using a structured-illumination epi-fluorescence microscope (ApoTome, Zeiss, Oberkochen, Germany) equipped with a Zeiss 20×/NA 0.40 Neofluar objective. A 3D stack of the autofluorescence of the chitin in the dioptrical apparatus was acquired. The eye curvature was measured by fitting a circular arc with radius *R* along the dorso-ventral and fronto-lateral directions. The distance between adjacent facets *D* was measured in the stack Z-projection and the morphological interommatidial angle was estimated as Δ*φ* = *D*/*R*. As the pseudopupillary pattern stays about constant under goniometric rotation of the eye, the morphological interommatidial angle is a good approximation for the optical interommatidial angle; for further details, see Belušič et al. (2013).

### Electrophysiological recordings

The spectral sensitivities of *Paysandisia*’s photoreceptors were measured by performing intracellular recordings in dark-adapted animals, using a high-impedance amplifier (SEC-10LX, NPI, Tamm, Germany) in bridge mode. The electrodes, pulled from borosilicate glass on a horizontal puller (P-97, Sutter, Novato, USA), filled with 3 M KCl, had a resistance in the range 50–100 MΩ. The reference electrode was a chloridized thin silver wire, inserted into the non-illuminated eye. The animals were positioned on a custom-made goniometer, and the light stimuli were provided by a photostimulator consisting of a 150 W xenon arc lamp, a monochromator (B&M Optik, Limburg, Germany), a shutter and a computer-controlled neutral density wedge filter (Thorlabs, Dachau, Germany). All optical elements were based on a UV-transmitting, fused-silica substrate. The linearly polarized stimuli, used for estimating the polarization sensitivity ratio (PSR) of the photoreceptors, were obtained by inserting a rotating UV-capable linear polarizer sheet (OUV2500, Knight Optical, UK) into the optical path.

Extracellular recordings yielding mass responses (electroretinogram or ERG) of the compound eyes and ocelli were performed with blunt borosilicate electrodes, filled with insect Ringer (0.67% NaCl, 0.015% KCl, 0.012% CaCl_2_, 0.015% NaHCO_3_, pH 7.2). The recording electrode was placed in the retina of the compound eye, at the edge of the illuminated patch. For the ocellar ERG, the recording electrode was placed adjacent to the ocellus. The compound eyes were selectively adapted using monochromatic light from LEDs with nominal peak wavelengths 380, 450, 525 and 625 nm (M3L1 series, 350 mA, Roithner LaserTechnik, Vienna, Austria), coupled to the main beam via a 50% fused-silica beam splitter. The spectral output of the LEDs was narrowed with a bandpass filter (UG11, Schott, Mainz, Germany) for the 380-nm LED and with long-pass filters (440, 500 and 600 nm) for the 450-, 525- and 625-nm LEDs. The response amplitudes to isoquantal monochromatic stimuli were calibrated against the intensity–response function to determine the effective stimulus intensities. The obtained spectral sensitivity profiles were fitted with a rhodopsin template (Stavenga et al. 1993; for further details, see Belušič et al. 2013; Ilić et al. 2016).

### Spectrophotometry

Reflectance spectra of different wing areas were measured with an AvaSpec 2048 CCD detector array spectrometer system (Avantes, Apeldoorn, the Netherlands), using a bifurcated probe (FCR 7UV200). The light source was a deuterium–halogen lamp (AvaLight D(H)S), and the reference was a white diffuse reflectance tile (WS2).

## Results

### Morphology of the eye

**Fig. 1:**
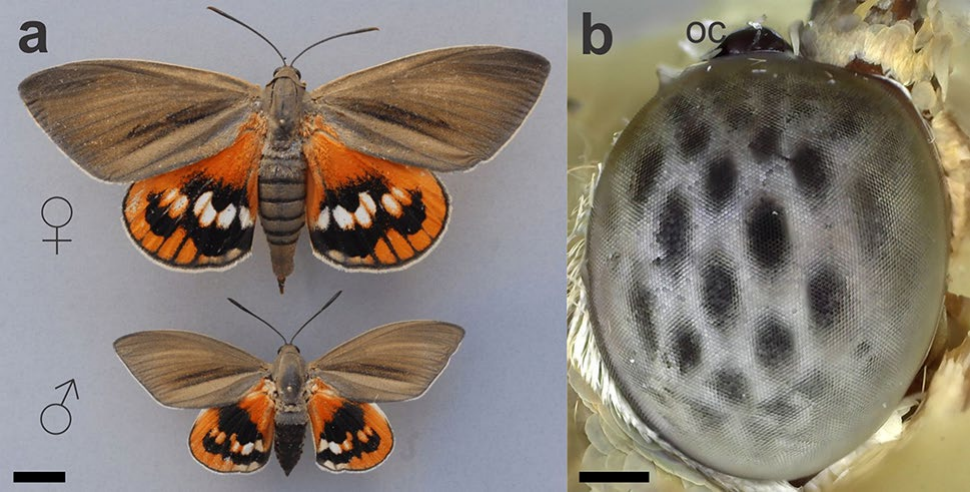
The giant butterfly-moth *Paysandisia* and its compound eye. **a** A female and a male *Paysandisia* as seen from the dorsal side. Photo courtesy of Dr. Jean-François Germain. **b** The compound eye featuring a conspicuous pattern of pseudopupils, and a large ocellus (oc). Scale bars: **a** 10 mm, **b** 0.5 mm

The male and female *Paysandisia archon* have a similar appearance. The female measures > 40 mm along the anteroposterior axis and > 90 mm across the wings; the male is distinctly smaller (Fig. 1a). The ellipsoidally shaped compound eyes (vertical diameter ~ 3.5 mm, horizontal diameter ~ 2.5 mm) consist of > 20,000 ommatidia. The facet diameter is about constant across the whole eye (~ 25 µm). We estimated the interommatidial angle Δ*φ* from the surface geometry of the eye, using a structured-illumination epi-fluorescence microscope (Fig. S1). The morphological interommatidial angle is less than a degree centrally (Δ*φ* = 0.8°) and almost doubles laterally (Δ*φ* = 1.4°). An ocellus is dorsally apposed to the compound eye (Fig. 1b). The compound eyes have a conspicuous pseudopupil pattern (Fig. 1b). When inspected with an epi-illumination telemicroscope, the eyes did not exhibit an eyeshine.

### Anatomy of the eye

**Fig. 2:**
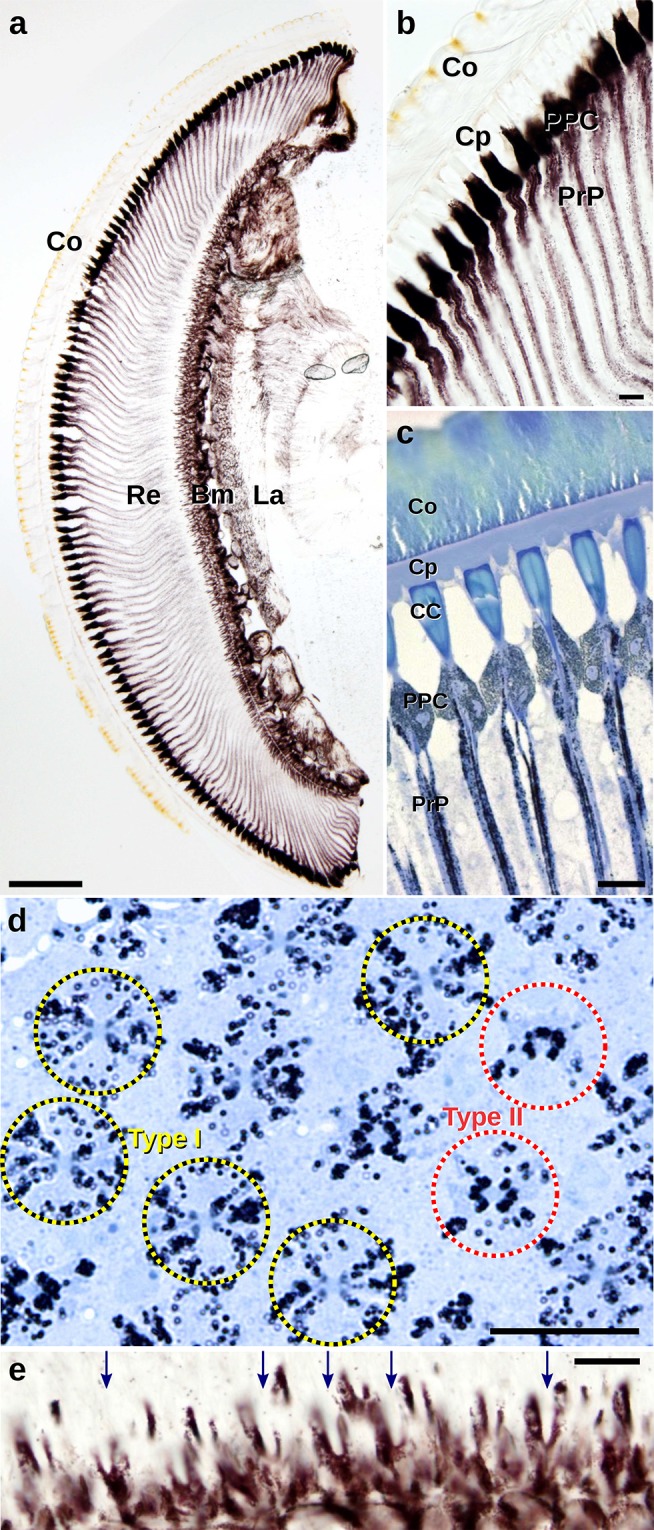
Light micrographs of histological sections of the central part of compound eye. **a** Longitudinal section in the dorsoventral direction with cornea (Co), corneal process (Cp), retina (Re), basal membrane (Bm) and lamina (La). **b, c** Longitudinal sections of the distal optical apparatus with the cornea (Co), corneal process (Cp), crystalline cone (CC), primary pigment cells (PPC), rhabdoms (R) and pigment granules in photoreceptors (PrP). **d** Dispersed pigments around type I distal rhabdom and pigments closely apposed to the type II rhabdom. **e** A magnified part of the basal retina with bilobed pigmented photoreceptor cells R9 (arrows). Scale bars: **a** 200 µm, **b**–**e** 20 µm

Light microscopic sections show that the compound eyes of *Paysandisia* are of the apposition type: the rhabdoms start close to the crystalline cone and the eyes have no clear zone (Fig. 2a–c). The dioptric apparatus occupies the distal 20% of the eye. It is composed of a ~ 50-µm-thick corneal facet lens, a prominent, ~ 12-µm-thick corneal process, and a funnel-shaped crystalline cone (diameter 11 µm, length 35–40 µm). The proximal 80% of the eye is taken up by the photoreceptor layer. The basement membrane is in the central part of the eye about ~ 600 µm below the corneal surface. The photoreceptor layer thickness is centrally ~ 500 µm, dorsally and ventrally ~ 400 µm (Fig. 2a).

The tips of the crystalline cones and the distal parts of the rhabdoms are sheathed by the densely pigmented primary pigment cells. The pigments seen proximally to the primary pigment cells reside in the photoreceptors (Fig. 2c, d). The region immediately distal to the basement membrane is also heavily pigmented (Fig. 2a), which is due to the pigment granules in the bilobed R9 photoreceptor cells (Fig. 2e).

Light microscopy (LM) demonstrated the presence of at least two ommatidial types (Fig. 2d). In a transverse section through the distal retina of an intermediately adapted eye (Fig. 2d), peri-rhabdomal pigment granules are found in two conformations, which we used to classify the ommatidia into two types. In type I ommatidia, the dark pigment granules are dispersed throughout the photoreceptor cell bodies, exposing four faint spots closely apposed to the rhabdom, which appear to belong to photoreceptor cells R5–8. In type II ommatidia, the pigment granules closely surround the rhabdom (Fig. 2d). The random distribution of the two types of ommatidia is seen in a larger cross section (Fig. S2). Type I ommatidia have dispersed pigment granules and four barely visible peri-rhabdomal spots, while type II ommatidia have four to six osmicated pigment clusters belonging to cells R3–8 closely apposed to the rhabdom. The two types of rhabdom occur randomly distributed in an approximate ratio I:II ≈ 1:2.

**Fig. 3:**
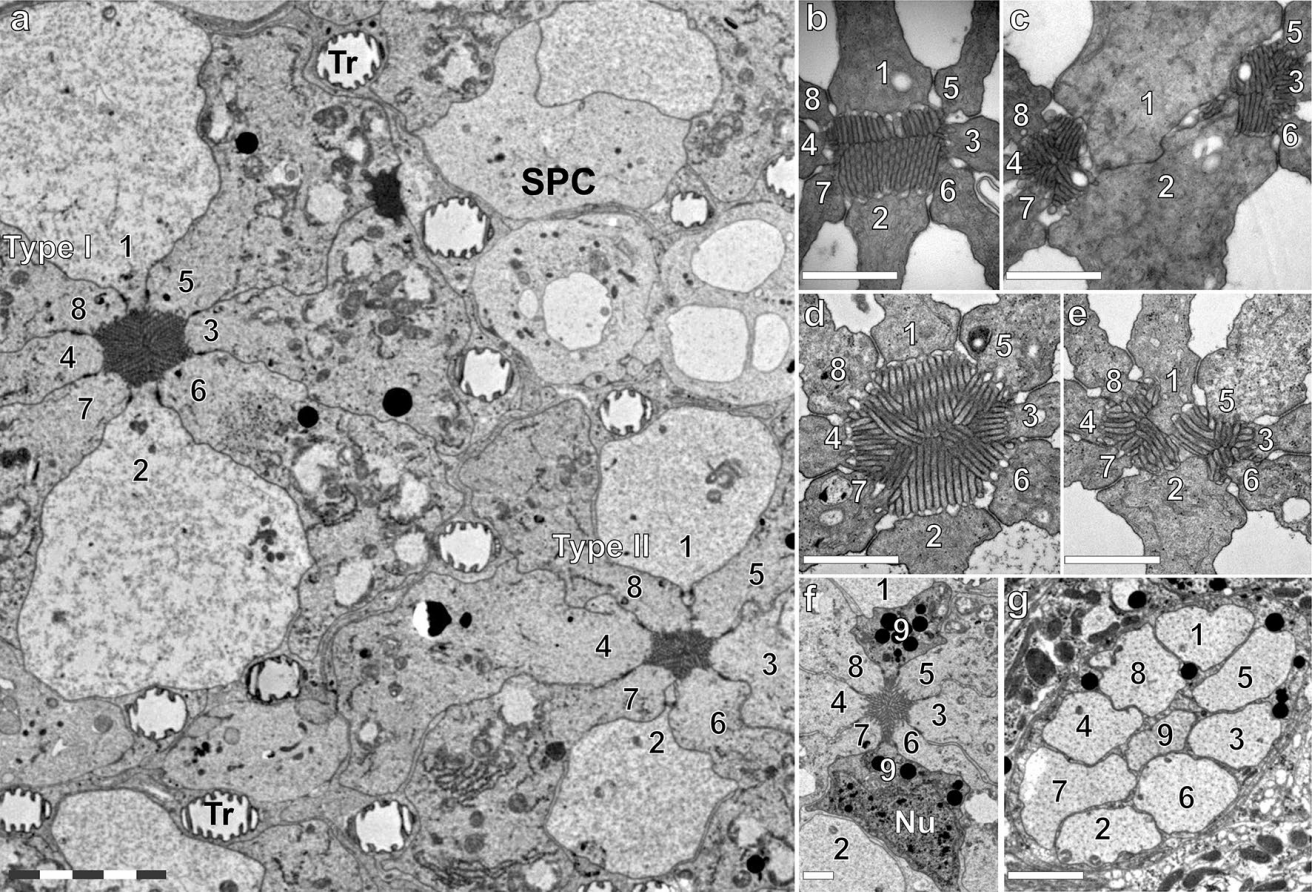
Transmission electron micrographs of the ommatidial types. **a** Type I and II rhabdoms at a proximal level, with numbered photoreceptors (1–8), secondary pigment cells (SPC) and tracheoles (TR); type I has a larger cross-sectional area than type II. In type I, the cell somata of receptors R1 and R2 retain contact with the rhabdom, while in type II the somata of R1 and R2 are pushed away from the rhabdom by the somata of the cells R5, R8 and R6, R7, respectively. **b** Distal section of type I ommatidium showing that the rhabdomeres of photoreceptors R1 and R2 are the major contributors to the rhabdom. **c** Distal section of type II ommatidium with the two spatially separated rhabdoms. The left sub-rhabdom receives microvillar contributions from cells R4, R7, R8. The right sub-rhabdom receives contributions from cells R3, R5, R6. Cells R1 and R2 contribute to both sub-rhabdoms. **d** The rhabdom proximally in a type I ommatidium with increased contributions of cells R3 and R8. **e** Fusion of the sub-rhabdoms proximally in the type II ommatidium. Contribution from cells R1–2 is minimal. **f** The basal cell R9 provides a semicircular pigmentary cushion at the bottom of the rhabdom and contributes a few microvilli. Its nucleus (Nu) is lateral to the rhabdomere. **g** Pseudocartridge of the nine axons of an ommatidium in the lamina below the basement membrane. Scale bars: **a** 5 µm, **b**–**g** 1 µm

We investigated the anatomy in more detail by performing transmission electron microscopy (TEM) in the eyes adapted to intermediate (indoor) light. The rhabdom of type I ommatidia is fused. Its size increases from distal (rectangular shape, 0.8 × 1.2 µm^2^, area ~ 1 µm^2^) to proximal (octagonal–round shape, diameter ~ 1.7 µm, area ~ 2.2 µm^2^; Fig. 3). The rectangular distal rhabdom consists predominantly of the rhabdomeres of the photoreceptors R1 and R2 (Fig. 3b). Medially, the rhabdom is composed of the microvilli of photoreceptor cells R1–8 (Fig. 3d). More proximally, the contribution of cells R1 and R2 is minor (Fig. 3a, f).

In type II ommatidia, the distal rhabdom is split into two oval sub-rhabdoms (principal diameters ~ 0.6 and ~ 1.0 µm, area ~ 0.55 µm^2^), spaced about 2 µm apart. Cells R1 and R2 contribute to both sub-rhabdoms, whereas cells R3, R5, R6 contribute only to one and R4, R7, R8 only to the other sub-rhabdom (Fig. 3c). The two sub-rhabdoms get somewhat smaller (area 0.4 – 0.5 µm^2^) and merge together about 350 µm proximally from the cornea (Fig. 3e). The contributions of cells R1 and R2 gradually decrease along the rhabdom until the cell somata lose contact with the rhabdom (Fig. 3a).

In both ommatidial types, immediately above the basement membrane, the densely pigmented and bilobed basal photoreceptor R9 adds a few microvilli to the rhabdom (Fig. 2a, e). Its nucleus is residing in one of the two lobes (Fig. 3f). Tracheoles are studded with small ribs (taenidia) and extend into the retinal layer (Fig. 3a), but there is no tracheolar basket close to the basement membrane (e.g. Kolb 1985). Consequently, no eyeshine is present, similarly as in Papilionidae and in contrast to most species from the other butterfly families (Stavenga 2002; Takemura et al. 2007). Pseudocartridges, formed by the nine photoreceptor axons from each ommatidium, penetrate the basement membrane (Fig. 3g).

### Morphological changes dependent on dark and light adaptation

**Fig. 4:**
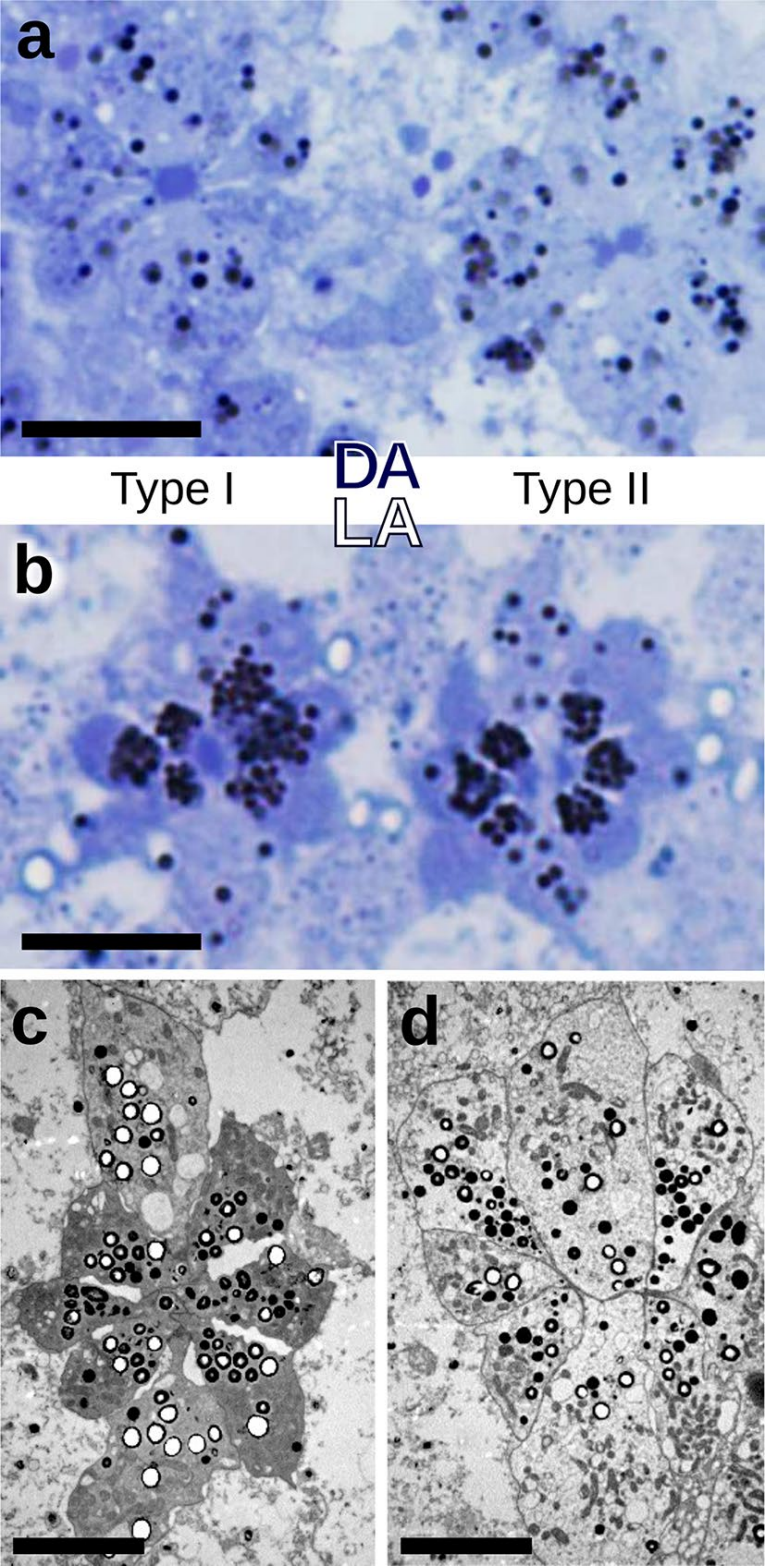
Structural changes occurring upon light adaptation. **a** Light micrograph of the distal part of two ommatidia in the dark-adapted (DA) state. Both in the type I (left) and type II ommatidium (right), pigment granules are dispersed throughout the somata of photoreceptor cells R3–8. **b** Light micrograph of the light-adapted (LA) state. The pigment granules are closely apposed to the rhabdom in the type I ommatidium (left) as well as to the semi-rhabdoms in the type II ommatidium (right). The photoreceptor cells R1 and R2 are less densely populated with granules than R3–8. **c**, **d** Transmission electron micrograph of the light-adapted ommatidia immediately below the crystalline cone showing the light-induced recession of the rhabdom in type I (**c**) and type II ommatidium (**d**). Scale bars: 10 µm

We investigated whether the eye morphology differs between the fully dark- and light-adapted states. Structural differences between dark- and light-adapted eyes could be easily identified (Fig. 4). In both type I and type II ommatidia of dark-adapted eyes, the pigment granules existing inside the photoreceptor somata were remote from the rhabdom (Fig. 4a), but in the light-adapted state, the granules were concentrated near the rhabdom (Fig. 4b). More surprisingly, whereas the rhabdom tips abutted the crystalline cones when dark adapted (Fig. S3), the rhabdoms had receded away from the crystalline cones in both ommatidial types when fully light adapted (Fig. 4c, d).

**Fig. 5:**
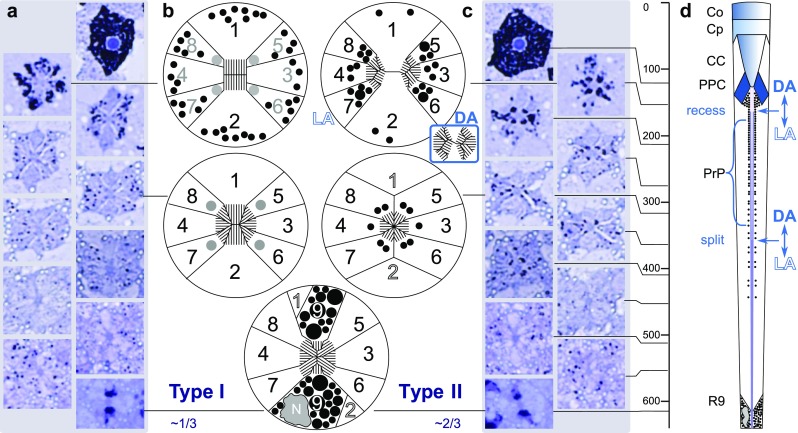
Schematic of the two ommatidial types. **a**, **c** Light microscopic cross sections of two ommatidia from both types (I and II) at the indicated depths. **b** Schematic cross sections at ~ 150 µm (top row), ~ 350 µm (mid row) and ~ 630 µm below the cornea. Photoreceptors that contribute microvilli to the rhabdom at each level are shown in black, those without the contribution in grey; R9 is bilobed and contains dense pigment granules. **d** Schematic longitudinal section of a type II ommatidium at the centre of the eye; the rhabdom is split distally and fuses at 350 µm below the cornea.* Co* corneal lens,* Cp* corneal process,* CC* crystalline cone,* PPC* primary pigment cells,* PrP* perirhabdomeral pigment,* R9* basal photoreceptor,* LA* light adapted state,* DA* dark adapted state

The two types of rhabdoms are schematically depicted in Fig. 5. A type I ommatidium and a type II ommatidium are tracked in LM sections from the cornea to the basement membrane (Fig. 5a, c). Distally and medially, the cells R1–8 that contribute the microvilli to the rhabdom are enumerated in black, the cells that do not contribute in grey and the cells without the contact to the rhabdom in outlined white (Fig. 5b). A longitudinal diagram for type II ommatidium is shown in Fig. 5d.

### Spectral sensitivity of the photoreceptors

**Fig. 6:**
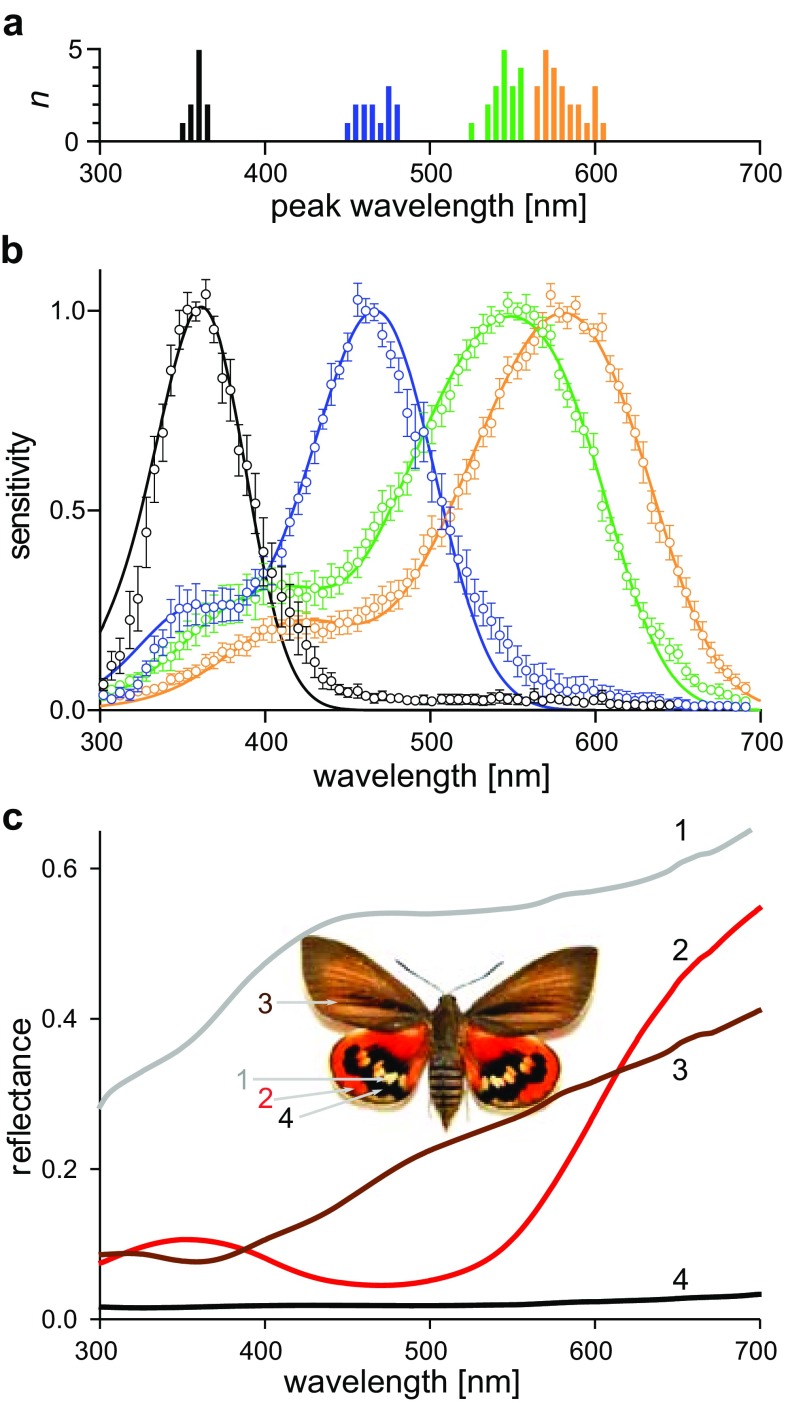
Spectral sensitivities of photoreceptors and reflectance spectra of the wings. **a** Histograms of the peak wavelength of the sensitivity spectra of the analysed photoreceptors (5 nm bins), distinguished into four classes: ultraviolet (UV), blue (B), green (G) and orange (O) cells (number of analysed units 10, 13, 18, 24, respectively). **b** Averaged spectral sensitivities of the four photoreceptor classes fitted with a rhodopsin template. Bars indicate standard error of mean. **c** Reflectance spectra of the wing areas, indicated in the inset

We investigated the photoreceptor spectral sensitivities by intracellular recordings. Out of > 100 recorded cells in six eyes (of four females and two males) we selected 64 stable recordings that yielded full spectral and intensity runs for further analysis. The different spectral types were encountered randomly in all preparations, so that the data from cells in both sexes were merged together. First, the spectral sensitivity of each cell was fitted with a rhodopsin template. The peak wavelengths of the fitted spectra appeared to cluster into four groups: UV, blue, green and orange (Fig. 6a). Clustering into the photoreceptor classes was objectively confirmed using principal component analysis of the sensitivity spectra (not shown). Subsequently, the average of the spectra of each group was fitted with a rhodopsin template (Fig. 6b). The resulting peak wavelengths for the four photoreceptor classes were 360, 460, 550 and 580 nm (Fig. 6b).

We investigated the polarization sensitivity in a few photoreceptors from each spectral class by presenting flashes of polarized light at the peak wavelength of the recorded photoreceptor. All tested cells had a moderate polarization sensitivity ratio (PSR ≈ 2).

### Extracellular recordings

To probe for possible additional spectral classes, we performed ERG recordings on the compound eye applying selective chromatic adaptation protocols. The ERG sensitivity curves could be well explained with a sum of the four rhodopsin templates obtained from the intracellular recordings (Fig. S4a). In other words, we did not obtain evidence for possible additional (e.g. violet or red) receptor classes. We also studied the spectral sensitivity of the ocelli by performing ERG recordings. The obtained sensitivity spectra had two peaks, in the UV and green, respectively, and could be unequivocally fitted with a sum of two rhodopsin templates having peak wavelengths at 350 and 550 nm, respectively (Fig. S4b).

### Wing colouration

The colours on *Paysandisia* wings presumably play a role in intraspecific recognition. We, therefore, measured wing reflectance spectra (Fig. 6c). The dorsal (upper) forewings are brown and the dorsal hindwings have a rich orange colour interrupted by black areas surrounding white spots (Fig. 1a). The reflectance spectrum of the orange wing patches has the steepest slope in the range 550–650 nm. Calculations of the wavelength discrimination with four photoreceptor classes (Vorobyev and Osorio 1998) indicate three ranges with potentially high spectral discrimination ability, around 410, 510 and 620 nm, in the violet-blue, green and orange-red ranges, respectively (Fig. S5). A detailed study on the colouration in *Paysandisia* is published elsewhere (Stavenga et al. 2018).

## Discussion

### A butterfly eye in a butterfly-moth?

The pseudopupil pattern in the *Paysandisia* eyes (Fig. 1b), visible with the naked eye, resembles that of e.g. nymphalid or pierid butterflies, suggesting that the eyes belong to the apposition type. The apposition was confirmed in our anatomical studies. We identified two ommatidial types with differently organized rhabdoms. The rhabdom of type I ommatidia has a larger cross section than the rhabdom of type II ommatidia. Both ommatidial types have nine photoreceptor cells. The photoreceptor tiering organization is similar to that found in the eyes of nymphalid butterflies (Swihart 1972; Gordon 1977; Kolb 1985), which, however, have three photoreceptor classes (Kinoshita et al. 1997). In type II ommatidia of *Paysandisia*, cells R1–8 contribute about equally to the distal rhabdom, while medially only cells R3–8 contribute. In type I ommatidia, cells R1–2 are the sole contributors to the distal rhabdom, contributing to about halfway the rhabdom (Fig. 3b, d). The predominance of cells R1–2 in the distal part of *Paysandisia*’s type I rhabdom has to our knowledge not been observed in butterflies; in nymphalids, the distal rhabdom is equally shared between cells R1–8 (Kolb 1985). The microvilli of the R1 and R2 photoreceptors in type I ommatidia are parallel, both distally and medially. The polarization sensitivity of photoreceptor cells was moderate (PSR ≈ 2) and comparable to that of other butterfly species (e.g. Bandai et al. 1992). While specialized ommatidial types containing photoreceptors with high PSR (> 10) do exist in dorsal rim areas (Labhart and Meyer 1999) and also in the central retina (Belušič et al. 2017; Heinloth et al. 2018), the moderate PSR found in the photoreceptors of general-purpose ommatidial types has been also shown to be the substrate for functional (i.e. not spurious) polarization vision (Wernet et al. 2012; Stewart et al. 2017). Thus, *Paysandisia* has the retinal substrate to potentially extract information from the polarized reflections from objects.

### Four photoreceptor classes, two ommatidial types

We identified four classes of spectral receptors. The measured sensitivity spectra conform well to rhodopsin templates. The low sensitivity of the ultraviolet receptor near 300 nm is likely due to the thick cornea acting as a UV-B-absorbing filter (Ilić et al. 2016). In selective adaptation measurements, the obtained ERG-based spectral sensitivity curves could be also well fitted with four visual pigment templates. We conclude that in *Paysandisia* the ancestral insect scheme of UV, blue and green photoreceptor classes is expanded by an additional long-wavelength (orange) receptor class, as was also found in *Spodoptera*, a noctuid moth with superposition eyes (Langer et al. 1979). Photoreceptor diversification is otherwise well known for diurnal butterflies (e.g. Frentiu et al. 2007; Marshall and Arikawa 2014; Chen et al. 2016; Arikawa 2017). In *Paysandisia*, the diversification with the green- and orange-peaking photoreceptor classes (Fig. 6b) enhances the theoretical colour discrimination ability in the green–red wavelength range (Fig. S5) which could be employed to enhance detection and discrimination of orange wing colours (Fig. 6c).

Based on the rhabdom morphology in different adaptation states, we identified two ommatidial types in *Paysandisia*. This contrasts with the studies on papilionids and pierids, which have demonstrated three morphologically distinct ommatidial types, with the photoreceptor pairs R1/R2 belonging to classes with UV/blue, UV/UV or blue/blue spectral sensitivities (Qiu et al. 2002; Chen et al. 2016; Perry et al. 2016; for reviews, see Wakakuwa et al. 2007; Stavenga and Arikawa 2011; Arikawa 2017). A similar scheme with three ommatidial types is present in sphingid moths (White et al. 2003), while in a saturnid moth, *Antheraea*, two ommatidial types (R1/2 blue/blue ventrally, UV/UV dorsally) have been suggested (Langer et al. 1986). According to the emerging comparative picture, we may predict that in *Paysandisia*, the R1/2 cells are either UV- or blue peaking, and the cells R3–8 are green and orange-peaking, but the exact allocation in distinct cells and ommatidial types remains to be elucidated. Further anatomical and electrophysiological analyses of the individual photoreceptors of *Paysandisia* may well reveal further diversification of the photoreceptor classes and the ommatidial types.

### Light adaptation mechanisms

We initially recognized the two ommatidial types due to the difference in the distribution of the pigment granules in the photoreceptor somata in histological sections of intermediately adapted eyes (Fig. 2d). The granules in type I ommatidia were dispersed, while those in type II ommatidia were aggregated near the rhabdom. Further adaptation experiments demonstrated that in both ommatidial types in the fully dark-adapted state the granules were dispersed, while in the fully light-adapted state they were all aggregated near the rhabdom (Fig. 4a, b). The mobile pigment granules in the photoreceptors probably function as a light-controlling pupil mechanism, similarly as in butterfly photoreceptors (Stavenga et al. 1977).

The dense pigmentation of the primary pigment cells likely reduces the off-axis incident stray light. A low amount of pigment granules in the medial part of the eye indicates that the light is well contained within the rhabdomeric waveguides. The function of the pigment in the bilobed basal R9 cells is to absorb the light reaching the bottom of the rhabdom. In the light-adapted ommatidia of both types, pigment granules migrate into the space between the crystalline cone and the receded rhabdom, likely reducing the light flux entering the rhabdom. Recently, a modelling study suggested that the pigment granules in the vicinity of the crystalline cone may also function to increase the wavefront matching with the rhabdom waveguide (Kim 2014).

In both ommatidial types, the rhabdoms abut the crystalline cones in the dark-adapted state. In the light-adapted state, however, the rhabdom tips recede about ~ 20 µm away from the crystalline cones. Changes in rhabdom composition have been observed during diurnal cycles in other arthropods (e.g. locusts: Williams 1982; grapsid crabs: Arikawa et al. 1987; desert ants: Narendra et al. 2013). Reshaping of the photoreceptors and their rhabdoms upon light and dark adaptation has been reported in some lepidopteran superposition eyes (Yagi and Koyama 1963; Horridge and Giddings 1971; Satoh et al. 2017), but to our knowledge not in the afocal apposition eyes of butterflies.

In type II ommatidia, the distal part of the rhabdom has a dumbbell-shaped cross section in the dark-adapted state, but upon light adaptation it splits into two separate sub-rhabdoms, each with diameter ~ 0.5 µm (Fig. 4a, b). In the light-adapted state, a substantial fraction of the light flux in the two slender rhabdom halves will propagate outside the rhabdom boundary. This reduces the light capture by the visual pigments (Vogt et al. 1982; Stavenga 2004) and increases the absorption by the surrounding pigment granules. The observed difference in the granule positions between the two ommatidial types in the intermediate state of light adaptation indicates that the pigment migration systems of the two ommatidial types have different operational ranges, perhaps somewhat analogous to vertebrate rods and cones (Fig. 2d, Fig. S2).

### Plasticity of the optical design of lepidopteran eyes

**Fig. 7:**
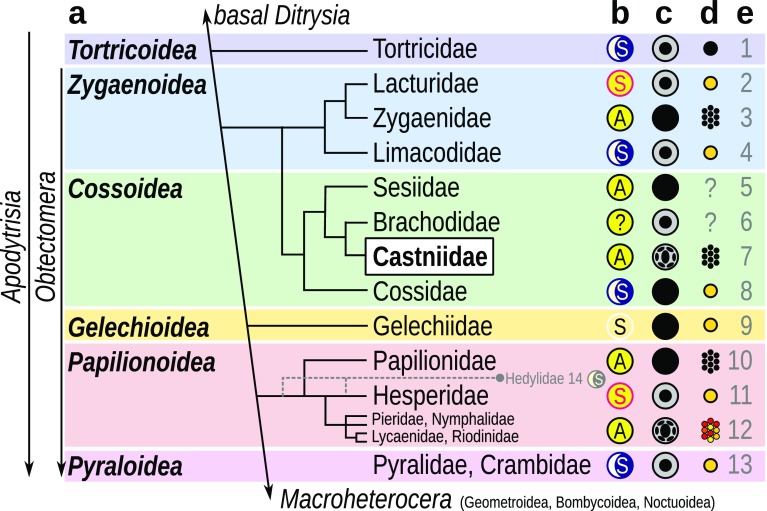
Visual system architectures in a part of the apodytrisian phylogeny. **a** Simplified phylogeny of the clade Apoditrysia (Mitter et al. 2017), showing a subset of six superfamilies (for details see Mutanen et al. 2010; Regier et al. 2013; Heikkilä et al. 2012, 2015). **b** Prevalent lifestyle in the group (sun: diurnal; moon: nocturnal) and eye architecture (S: superposition; A: apposition). **c** External appearance of the eye with base colour and the pseudopupillary pattern. **d** Eyeshine: black circle: superposition without eyeglow; yellow circle: superposition with eyeglow; colourful hexagonal lattice: apposition eyeshine; black hexagonal lattice: apposition but no eyeshine. **e** Common group names, studied species, and relevant references: **1** leaf roller moths (*Adoxophyes*: Satoh et al. 2017); **2** tropical burnets; **3** burnets (Yagi and Koyama 1963); **4** slug moths (Yagi and Koyama 1963); **5** clearwing moths (*Synanthedon*: Eby et al. 2013); **6** little bear moths; **7** giant butterfly-moths and sun moths (*Paysandisia*: this study); **8** carpenter and goat moths; **9** curved horn moths; **10** swallowtail butterflies (*Papilio xuthus*: Arikawa et al. 1999; Kitamoto et al. 1998; Koshitaka et al. 2008; *Papilio glaucus*: Briscoe 1998, *Graphium*: Chen et al. 2016); **11** skipper butterflies (*Parnara*: Shimohigashi and Tominaga 1986; *Ocybadistes*: Land 1984); **12** whites and yellows (*Pieris*: Arikawa et al. 2005; *Colias*: Ogawa et al. 2012, 2013); brushfoots (*Aglais, Vanessa*: Briscoe and Barnard 2005; *Sasakia*: Kinoshita et al. 1997), blues, coppers and sunbeams, metalmarks (*Lycaena, Polyommatus*: Sisson-Mangus et al. 2008); **13** snout moths (Pyralidae: *Ephestia*: Cleary et al. 1977), grass moths (Crambidae: *Ostrinia*: Belušič et al. 2017); **14** moth-butterflies (Hedylidae: *Macrosoma*: Yack et al. 2007). Reviews: Stavenga 2002, Arikawa and Stavenga 2006, Wakakuwa et al. 2007; Arikawa 2017)

Castniidae have a curious geographical distribution. The giant butterfly-moths are native to South and Central America, and the smaller sun moths are native to Australia and Southeast Asia (O’Dwyer and Attiwill 1999; Braby and Dunford 2006; Sarto i Monteys et al. 2016). Photographs of live specimens show multiple pseudopupils, indicating that both family branches have apposition eyes. Despite the long-term separation of Australia from Gondwana, both branches retain traits that are associated with a diurnal lifestyle, e.g. clubbed antennae and colourful wings (Heikkilä et al. 2015). A simplified phylogeny, shown in Fig. 7a (based upon Mutanen et al. 2010; Regier et al. 2013; Heikkilä et al. 2015; Mitter et al. 2017), is annotated with diurnal/nocturnal lifestyle, eye architecture, pseudopupil pattern and presence of the eyeshine (Fig. 7b–d). Castniidae belong to the superfamily Cossioidea in the clade Apodytrisia. Their exact phylogenetic position is not fully resolved. They are either just outside of the subclade Obtectomera, or at its start, with the Papilionoidea being put towards its end (Heikkilä et al. 2015). Castniidae are closely related to the diurnal clearwing moths (Sesiidae) with apposition eyes (Eby et al. 2013) and the nocturnal carpenter moths (Cossidae) with superposition eyes. In the related superfamily Zygaenoidea, the diurnal burnets (Zygaenidae) have apposition eyes (Yagi and Koyama 1963), while the two other families (Lacturidae, Limacodidae) may have superposition eyes. A transition in the optical design—either from superposition to apposition or vice versa—has possibly happened separately within these two superfamilies. The eye design transition has also happened once or twice in Papilionoidea (see Fig. 7), and perhaps several times within the last (and largest) lepidopteran subclade, the “eared moths” Macroheterocera. A transition from superposition to* afocal* apposition seems to be achieved easily in terms of evolution, as it requires only a redistribution of pigment granules and a modest change of the dimensions of the lens–cone system (Nilsson et al. 1988). It is intriguing that the apposition eyes of *Paysandisia* seem to be of the afocal type, as in the diurnal butterfly families. This may have further implications for understanding the eye design evolution within Lepidoptera, as discussed by Nilsson et al. (1988) and Fischer et al. (2012, 2014).

We have found that *Paysandisia* has nine photoreceptor cells per ommatidium. The basal photoreceptor R9 is bilobed, with the nucleus residing in one of the lobes. In the apposition eye of the closely related clearwing moths (Sessidae), a similar scheme with eight distal cells and a basal pigment cell was reported, based on light microscopy (Eby et al. 2013). The basal group Tortricidae has seven distal photoreceptors and one axial basal photoreceptor, which is not bilobed (Satoh et al. 2017). Papilionoidea have nine photoreceptors per ommatidium, except for the Hedylidae which have eight photoreceptors (seven distal and one proximal; Yack et al. 2007). Within the Apodytrisia, higher numbers (more than ten) of photoreceptors per ommatidium have been confirmed in Pyraloidea (Horridge and Giddings 1971; Belušič et al. 2017). To our knowledge, Castniidae are the most basal group in the lepidopteran subclade Apodytrisia where an ultrastructural study confirmed the presence of nine retinula cells per ommatidium and a bilobed basal cell with a paraxial nucleus. The paraxial and axial positioning of the nuclei of the basal cells are the morphological correlates of the apposition and superposition eye designs, respectively (Fischer et al. 2012), further supporting the finding that *Paysandisia* has compound eyes of the apposition type.

### Rhabdom secession: an asset with afocal optics?

In the focal apposition eyes of e.g. Diptera, Hymenoptera, the rhabdom must start close to the focal plane of the dioptric apparatus for the acceptance angles to remain small (Land et al. 1999; Stavenga 2003). In the afocal apposition eyes of butterflies, the position of the rhabdom start is not critical (Nilsson et al. 1988). The dimensions of the dioptric apparatus and the presence of the corneal process, along with the ease of transition between superposition and afocal apposition all suggest afocal optics in *Paysandisia*. We hypothesize that in this case the retraction of the rhabdom away from the dioptric apparatus might cause a reduction (sharpening) of the acceptance angle. This prediction can be inferred from geometric ray tracing (e.g. Fig. 6 in Nilsson et al. 1988), but should be modelled with a wave propagation model (e.g. Kim 2014) and put to an experimental test. Acceptance angle sharpening in the light-adapted state has been demonstrated in butterflies and explained via selective absorption of higher order waveguide modes (Land and Osorio 1990).

## Conclusion

The palm borer moth *Paysandisia archon* is a large diurnal insect with apposition eyes. The three described retinal light adaptation mechanisms (pigment migration, rhabdom splitting and retraction) provide a wide dynamic operating range and an increased acuity in bright light conditions. The two described morphological ommatidial types may be optimized for different operational light sensitivity ranges. The visual system is presumably tuned towards finding mating partners: the additional long-wavelength photoreceptor class may serve to enhance detection of wing colours. The variability in the optical eye design in the families closely related to the butterfly-moths and sun moths proves the remarkable evolutionary plasticity of lepidopteran eyes.

## Electronic supplementary material

Below is the link to the electronic supplementary material.

Supplementary material 1 (PDF 1165 KB)
