# Supplementary material for: The giant butterfly-moth Paysandisia archon has spectrally rich apposition eyes with unique light-dependent photoreceptor dynamics
Source: J Comp Physiol A Neuroethol Sens Neural Behav Physiol. 2018 Jun 4;204(7):639–51. doi: 10.1007/s00359-018-1267-z (PMC6028894; doi:10.1007/s00359-018-1267-z)
Supplement: Supplementary file 1 — Supplementary material 1 (PDF 1165 KB) [file 359_2018_1267_MOESM1_ESM.pdf]

The giant butterfly-moth *Paysandisia archon* has spectrally rich apposition eyes with unique light-dependent photoreceptor dynamics

Primož Pirih, Marko Ilić, Jerneja Rudolf, Kentaro Arikawa, Doekele Stavenga, Gregor Belušič

Supplementary material

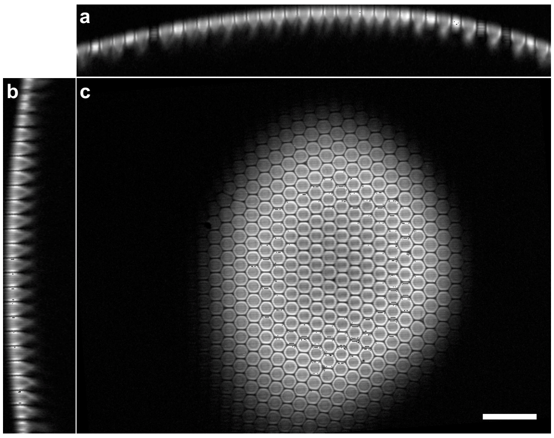

Fig. S1. Geometry of the compound eye of *Paysandisia archon*; autofluorescence of chitin in the dioptrical apparatus (**a**) Fluorescence micrograph obtained with a Zeiss Apotome2, showing a transverse section through the center along the horizontal coordinate. (**b**) A transverse section through the center along the horizontal coordinate. (**c**) A face-on view of the compound eye showing the perfect lattice of the facet lenses. The sections have been used to estimate the interommatidial angle. The facet lens diameters D were measured and a circle was fitted to the transverse sections to obtain the radius of curvature, R. The morphological interommatidial angle was calculated as $\Delta\phi = D/R$.

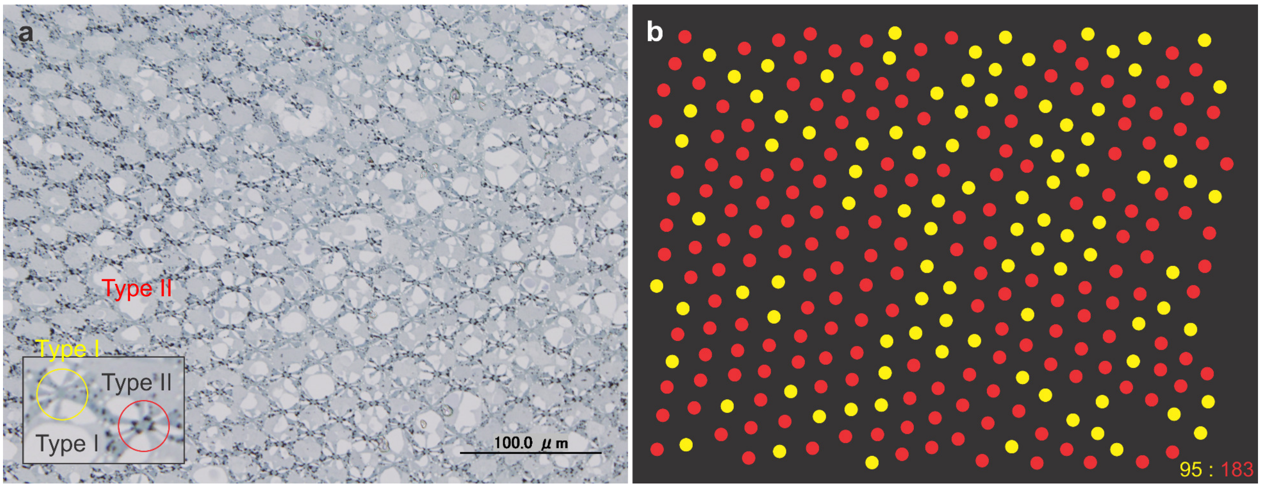

Fig. S2. The two ommatidial types in a restricted part of the eye. (**a**) A light microscopic section with type I and type II indicated by a yellow and red circle. (**b**) A distribution map of the two ommatidial types indicated by a filled yellow and red circle. Missing circles in the lattice signify ommatidia whose type could not be determined.

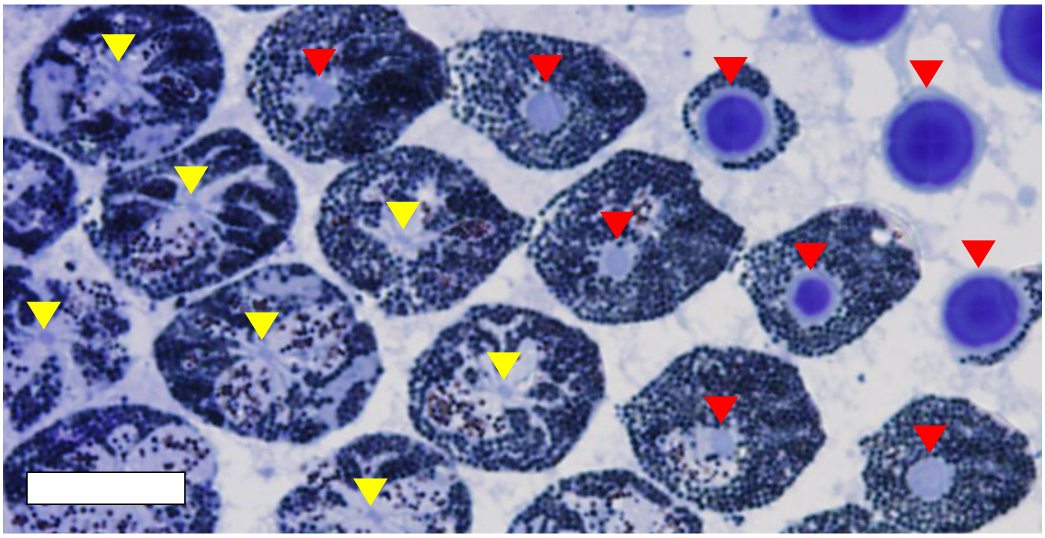

Fig. S3. Light micrograph of an oblique section of a dark-adapted distal retina. Rhabdoms (yellow triangles) contact the tips of crystalline cones (red triangles). Scale bar, 20 µm.

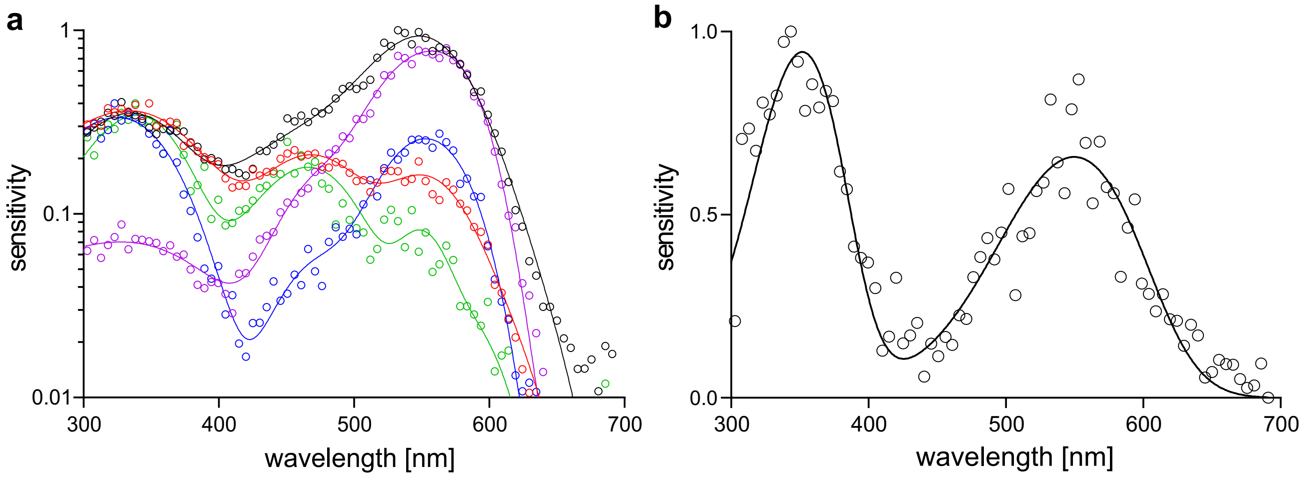

Fig. S4. Spectral sensitivities of the compound and ocelli eyes as measured by electroretinography (ERG) and stimulation with monochromatic light. (**a**) The dark-adapted compound eyes (black curve) have two main sensitivity bands in the UV and green wavelength range, but an additional shoulder is present in the blue wavelength range. By selective adaptation with red (red curve, 625 nm LED) or green light (green curve, 525 nm LED), sensitivity in the green is reduced and the blue shoulder turns into a distinct blue band; adaptation with blue light (blue curve, 450 nm LED) suppresses sensitivity in the blue and less in the green; adaptation with UV light (pink curve, 380 nm LED) suppresses sensitivity in the UV. The curves represent linear sums of the four rhodopsin templates with peak wavelengths, determined with intracellular recordings (peaks at 360, 465, 550, 580 nm). The spectral data could be well fitted also with the sum of three opsins (360, 465, 550 nm; not shown), indicating that using selective adaptation, it is not possible to isolate the 580 nm (orange) spectral class. (**b**) Spectral sensitivity of an ocellus, showing two main sensitivity bands, in the UV and green wavelength range, fitted with a sum of rhodopsin templates (350, 540 nm).

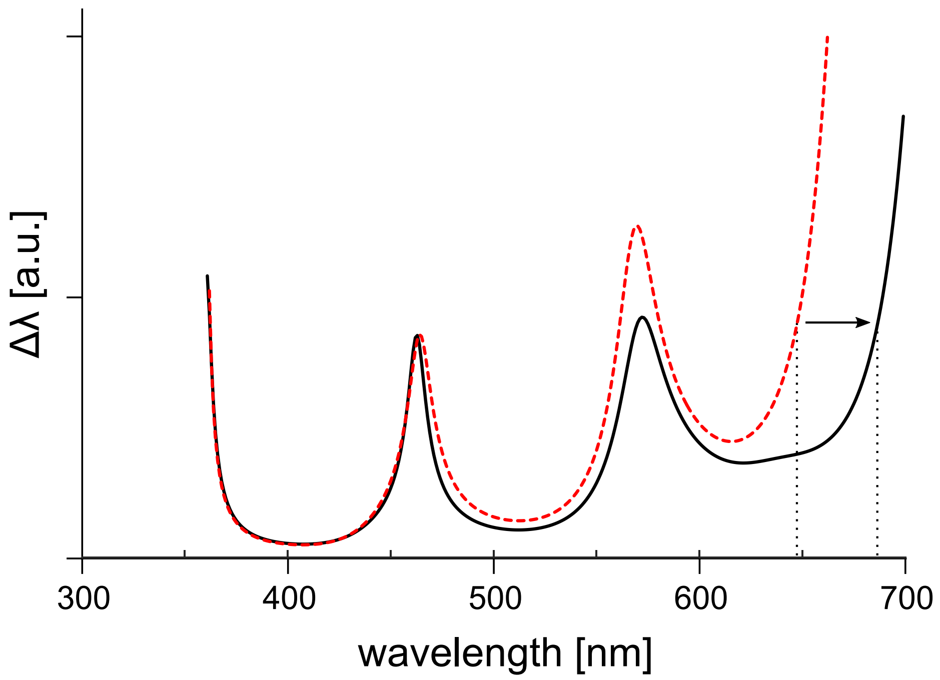

Figure S5. Wavelength discrimination calculated with the sensitivity spectra of Fig. 6b, using the Vorobyev-Osorio formalism*. The relative discrimination ability is calculated based on the model with three receptor classes peaking in the UV, blue and green (360, 465, 550 nm; *red dashed line*), and based on the model with all four receptor classes, including the orange class peaking at 580 nm (*black solid line*). The two curves were scaled to match in the range 380-470 nm. The four receptor class model extends the theoretical discrimination ability in the long wavelength range for about 35 nm (*arrows, dotted vertical lines*).

*Vorobyev M, Osorio D (1998) Receptor noise as a determinant of colour thresholds. Proc R Soc Lond B 265:351–358
